# Supplementary material for: Magnitude of Hepatitis B virus and associated factors among chronic liver disease patients admitted to the medical ward at Sheikh Hassen Yabare Comprehensive Specialized Hospital, Jigjiga, Ethiopia: a retrospective study
Source: PLoS One. 2025 Dec 30;20(12):e0338702. doi: 10.1371/journal.pone.0338702 (PMC12752937; doi:10.1371/journal.pone.0338702)
Supplement: S2 File — (DOCX) [file pone.0338702.s002.docx]

## Annex

## Data Collection Abstraction Sheet

Data collection abstraction sheet to assess the magnitude of HBV and Associated Factors among Chronic Liver Disease Patients Admitted to the Medical Ward at Sheikh Hassen Yabare Comprehensive Specialized Hospital, Jigjiga, Ethiopia

Instructions: Tick/write the code corresponding to the answer to each question.

Date of the checklist filled (dd/mm/yyyy): ____/__/___/___/___

| **1: Background information** | 1.1 Code |  | |
| --- | --- | --- | --- |
|  | 1.2 Sex |  | |
|  | 1.3 Age |  | |
|  | 1.4 Residency | Urban |  |
|  |  | Rural |  |
| **2:Questions related to HBV risk factors** | 2.1 Ear Piercing | Yes |  |
|  |  | No |  |
|  | 2.2dental extraction | Yes |  |
|  |  | No |  |
|  | 2.2 Nose piercing  . | Yes |  |
|  |  | No |  |
|  | 2.4 Contact with a jaundiced patient | Yes |  |
|  |  | No |  |
|  | 2.5 Surgical procedure | Yes |  |
|  |  | No |  |
|  | 2.6 Abortion | Yes |  |
|  |  | No |  |
|  | 2.7 Hospital admission | Yes |  |
|  |  | No |  |
|  | 2.8 multiple sexual partner | Yes |  |
|  |  | No |  |
|  | 2.9 history of STI | Yes |  |
|  |  | No |  |
|  | 2.10 Blood transfusion | Yes |  |
|  |  | No |  |
| **3:Hbv status** | Yes | | |
|  | No | | |

## 
