# Supplementary material for: Magnitude of Hepatitis B virus and associated factors among chronic liver disease patients admitted to the medical ward at Sheikh Hassen Yabare Comprehensive Specialized Hospital, Jigjiga, Ethiopia: a retrospective study
Source: PLoS One. 2025 Dec 30;20(12):e0338702. doi: 10.1371/journal.pone.0338702 (PMC12752937; doi:10.1371/journal.pone.0338702)
Supplement: Attachment — Submitted filename: Review.docx [file pone.0338702.s003.docx]

**Review**

This review assesses the manuscript "Magnitude of Hepatitis B virus and associated factors among chronic liver disease patients admitted to the medical ward at Sheikh Hassen Yabare Comprehensive Specialized Hospital, Jigjiga, Ethiopia" against the PLOS One publication criteria.

**Review Against PLOS One Publication Criteria:**

1. **The study presents the results of original research.** The manuscript clearly states that data on the proportion of HBV and its associated factors among chronic liver disease patients is scarce in the study area and in Ethiopia at large. The objective is to assess this magnitude and associated factors at Sheikh Hassen Yabare Comprehensive Specialized Hospital, indicating original research addressing a specific knowledge gap.
2. **Results reported have not been published elsewhere.** While the document itself does not explicitly state that the results have not been published elsewhere, it is presented as a manuscript for submission to PLOS One. The presence of sections for financial disclosure and competing interests, typical for new submissions, suggests it is an original, unpublished work.
3. **Experiments, statistics, and other analyses are performed to a high technical standard and are described in sufficient detail.** The methodology is well-described, outlining an institutional-based retrospective cross-sectional study with 249 participants selected by simple random sampling. Data collection utilized Kobo Toolbox software, and analysis was performed using SPSS version 29.0, employing binary logistic regression. The authors detail checks for model fitness using the Hosmer-Lemeshow goodness-of-fit test and multicollinearity using the variance inflation factor. Statistical significance was set at P<0.05, and adjusted odds ratios with 95% confidence intervals were used to measure association strength. The sample size calculation and data quality control measures, including pre-testing and daily completeness checks, are also described.
4. **Conclusions are presented in an appropriate fashion and are supported by the data.** The conclusions align directly with the reported results. The study concludes that the magnitude of HBV among clinically diagnosed chronic liver disease patients was high and that male sex, history of hospital admission, blood transfusion, and contact with jaundiced individuals were significantly associated with HBV. These conclusions are directly supported by the presented AORs and 95% CIs in the results section (e.g., AOR=3.60 for hospital admission, AOR=2.22 for blood transfusion, AOR=2.65 for male sex, AOR=4.52 for contact with jaundiced individuals).
5. **The article is presented in an intelligible fashion and is written in standard English.** The manuscript is generally clear, well-structured, and intelligible. It follows standard scientific writing conventions, making it easy to understand the background, methods, results, and conclusions. Minor grammatical imperfections exist (e.g., "male sex"), but they do not impede comprehension.
6. **The research meets all applicable standards for the ethics of experimentation and research integrity.** The authors explicitly state that ethical approval was obtained from the Jigjiga University Research and Ethics Review Board (JJURERB0056/2024). Permission was also acquired from the hospitals to extract data from patient medical records, with all patient data anonymized and held with strong confidentiality. This indicates adherence to ethical standards for human participant research.
7. **The article adheres to appropriate reporting guidelines and community standards for data availability.** The manuscript includes a clear data availability statement indicating that "all data are fully available without restriction" and are "within the manuscript and its Supporting Information files". This complies with typical community standards for data availability in research publications
