# Supplementary material for: Magnitude of Hepatitis B virus and associated factors among chronic liver disease patients admitted to the medical ward at Sheikh Hassen Yabare Comprehensive Specialized Hospital, Jigjiga, Ethiopia: a retrospective study
Source: PLoS One. 2025 Dec 30;20(12):e0338702. doi: 10.1371/journal.pone.0338702 (PMC12752937; doi:10.1371/journal.pone.0338702)
Supplement: Attachment — Submitted filename: Response to Reviewers (4).docx [file pone.0338702.s007.docx]

**Reviewers' comments:**

**Comments to the Author**

**Reviewer #1:**

The methods section, while comprehensively detailing the statistical analyses and ethical considerations, could benefit from a deeper elaboration on certain aspects to enhance transparency and reproducibility. Specifically, while a "lottery method" for simple random sampling is mentioned, more granular detail on its practical execution could be provided to ensure clarity for replication. Additionally, given the retrospective nature of the study, a brief acknowledgment within the methods about the inherent limitations of using pre-existing medical records, such as potential data inconsistencies or missing information, could add a layer of robustness. Finally, while software versions are cited, further specifics on the implementation within tools like Kobo Toolbox (e.g., form validation, data cleaning protocols) or custom scripts used in SPSS, beyond standard regression techniques, would enrich the methodological description.

**Reviewer's Responses:**

Dear reviewer, thank you for your comments and suggestions:

- We revised the ‘lottery method’ into computer computer-generated method to select the study participants in both the abstract and method sections.
- We added that the retrospective nature of the records can lead to missing data, and we incorporated it in the limitation section of the manuscript.
- We used Kobo Toolbox for the validation and cleaning of the data
- We used custom syntax for data re-coding and diagnostics

**Reviewer #2**: Line 186: The formula uses a prevalence rate (P) of 20.4%, but no source is cited. Please add a reference. Lines 304-306: The claim that the current result (32.53%) is lower than the Hawassa study (79.5%) is attributed to "increased vaccination coverage." However, no local vaccination data is provided to support this inference. It is recommended to add a citation referencing literature on Ethiopia's hepatitis B vaccination rate.

**Reviewer's Responses:**

Dear reviewer, we are grateful to get these insightful comments and suggestions:

- We have cited the study from which we took the proportion to calculate the sample size.
- We have cited the paragraphs on the lines you quoted
- We have revised the justification for the discussion section based on your question and suggestion.

**Editor’s comments**

| - Title should be revised as: Magnitude of Hepatitis B virus and associated factors among chronic liver disease patients admitted to the medical ward at Sheikh Hassen Yabare Comprehensive Specialized Hospital, Jigjiga, Ethiopia: a retrospective study | - Thanks, dear editor, we have revised per your suggestion |
| --- | --- |
| - A thorough revision of the English language is needed. The writing lacks fluency and has serious issues with grammar that need to be addressed. The document suffers from major coherence problems. It requires an intensive revision in all aspects. | - We revised as per your recommendation |
| - HBV: It should be written in both forms at least once as ‘Hepatitis B virus (HBV)’ | - We revised based on your recommendation |
| - Lines 33 &34: A total of 249 study subjects were selected by…… It should be written as; A total of 249 study participants was included………………. | - Dear editor, we have revised based the suggestion |
| - Which laboratory technique/method was employed to diagnose HBV? It should be mentioned both in the abstract and the methods part. | - We have incorporated the method of laboratory investigation of the participants. |
| - In the abstract and result, the absolute number (numerator and denominator) is needed together with the percentage. For example, A/B (C %). | - Thanks for the input, we have included the numbers as well as the percentage in the abstract and the results |
| - Your conclusion in the abstract part should be supported by evidence. It was beyond your scope and requires revision. | - We have revised based on the main findings |
| - Line 153: Materials and methods | - We have revised based on the suggestion |
| - The laboratory method should be clearly stated in the methods part, even though retrospective. | - We have mentioned the laboratory methods both in the abstract and the method section |
| - Line 166: Study design and period | - Dear editor, we have reshuffled based on the suggestion |
| - The inclusion and exclusion criteria should be placed separately and clearly. | - We have separated the inclusion and the exclusion criteria based on the given suggestion |
| - Line 182: The sample size for the first specific objective was calculated using a single population… | - We have revised based on the suggestion |
| - Line 184: The formula should be prepared by word text, not figure. | - We prepared it in text form and revised accordingly |
| - Discussion not merely a comparison, but it includes justifications of your results | - We have revised based on your comments and suggestions |
| - Your conclusion and recommendation were too ambitious. It should be specific and based on your results. | - We revised it based on the main findings |
| - Figures and tables should be prepared to the standard. | - We have revised based on the given suggestion |
| - The tables can be inserted where you cited within the main manuscript/text. | - We acknowledge your suggestions and inserted the tables into the main manuscript/text |
| - Unnecessary bolds should be avoided from the ‘conclusion’ part. The recommendation seems specific to concerned body. Please write in a paragraph form. | - We appreciate your concerns, and we have amended based on the suggestions |
| - The references were not written properly. All references should be written correctly according to Vancouver style. | - We made all the references Vancouver style |
| - Follow the standard binomial nomenclature, italize journal name and the word ‘et al’ | - Dear editor, we have revised based on the suggestion. |
| - NB: follow the guidelines for manuscript writing protocol for PLoS One. | - Well noted |
