# Supplementary material for: Magnitude of Hepatitis B virus and associated factors among chronic liver disease patients admitted to the medical ward at Sheikh Hassen Yabare Comprehensive Specialized Hospital, Jigjiga, Ethiopia: a retrospective study
Source: PLoS One. 2025 Dec 30;20(12):e0338702. doi: 10.1371/journal.pone.0338702 (PMC12752937; doi:10.1371/journal.pone.0338702)
Supplement: Attachment — Submitted filename: Reviewed Manuscript.docx [file pone.0338702.s005.docx]

**Magnitude of Hepatitis B virus and associated factors among chronic liver disease patients admitted to the medical ward at Sheikh Hassen Yabare Comprehensive Specialized Hospital, Jigjiga, Ethiopia: a retrospective study.**

Ramadan Budul Yusuf ^1,^ Getnet Addisu^2^**,** Ahmed Mohammed Ibrahim^1 3 4^, Terefe Gebre^2^ seid Muhumed Abdilahi^5,^ Bilan Ali Ahmed^6^, Abdulahi Hussein Abdinur^2^, Girma Tadesse^1^, Mohamed Omar Osman^1^

^1^ [Department of Public Health, Institute of Health Science, Jigjiga University, Jigjiga, Ethiopia].

^2^[School of Medicine, Institute of Health Science, Jigjiga University, Jigjiga, Ethiopia].

^3^[Swiss Tropical and Public Health Institute, Kreuzstrasse 2, 4123, Allschwil, Switzerland

^4^[University of Basel, Petersplatz 1, 4003, Basel, Switzerland].

^5^[Department of Pediatrics and child health Nursing, Institute of Health Science, Jigjiga University, Jigjiga, Ethiopia].

^6^[Department of Midwifery, Institute of Health Science, Jigjiga University, Jigjiga, Ethiopia].

Correspondence: *[Ramadan Budul Yusuf]

[P.O.BOX: 1020, Jigjiga, Ethiopia]

Tel [+251915468383]

Fax [+251 (0) 25 775 5976]

Email:ramadanbudul83@gmail.com/ramadan.budul@jju.edu.et

# ABSTRACT

**Background**: Viral hepatitis is still a major public health problem around the globe, and acquiring adequate and recent epidemiological data on Hepatitis B is important in the prevention and control of the disease. Data on Hepatitis B virus (HBV) among patients with chronic liver disease are scarce in the study area, as well as in Ethiopia at large.

**Objective:** To assess the magnitude of Hepatitis B virus (HBV) and its associated factors among chronic liver disease patients admitted to the medical ward at Sheikh Hassen Yabare Comprehensive Specialized Hospital, Jigjiga, Ethiopia.

**Method:** An institution-based retrospective cross-sectional study was conducted in the Sheikh Hassen Yabare Comprehensive Specialized Hospital. A total of 249 study participants were selected by simple random sampling using a computer-generated method. A rapid test of HbsAg using a kit from Henso Medical (HANGZHOU) Co. Ltd. was used. The data were collected using Kobo toolbox and analyzed using SPSS version 29.0. Binary logistic regression was employed to identify statistically significant factors for HBV. Model fitness was checked by the Hosmer-Lemeshow goodness-of-fit test, and multicollinearity across variables was checked using the variance inflation factor. Statistical significance was considered at P<0.05, and an adjusted odds ratio with a 95% confidence interval was used to measure the strength of the association.

**Result:** The magnitude of HBV among clinically diagnosed chronic liver disease patients was 81/249 (32.53%) (95%CI = 26.9–39.0). Having a history of hospital admission (AOR = 3.60, 95% CI = 1.84–7.04), blood transfusion history (AOR = 2.22, 95CI = 1.13–4.37), being Male sex (AOR = 2.65, 95CI = 1.18, 5.96) and a history of contact of jaundiced individuals (AOR = 4.52, 95CI = 2.34, 8.72) were also significantly associated with HBV.

**Conclusion:** The magnitude of HBV among clinically diagnosed chronic liver disease patients was high. Being male sex, having a history of hospital admission, blood transfusion, and contact history with jaundice patients was significantly associated with HBV. The hospital should strictly follow blood transfusion safety protocols and implement proper infection control measures to minimize HBV transmission. Additionally, healthcare providers should screen patients with a history of hospital admission, contact with jaundice patients to facilitate early diagnosis and treatment.

**Keywords**: hepatitis B virus, chronic liver disease, Sheikh Hassen Yabare Comprehensive Specialized Hospital

# INTRODUCTION

A liver inflammation is generally referred to as hepatitis. It happens as a result of autoimmune illnesses, alcoholism, drug exposure, chemical exposure, and toxin exposure, among other pathogen infections(1). Through immunological anergy, the hepatotropic virus known as the hepatitis B virus (HBV) can cause a chronic and persistent infection in humans (2).

Covalently closed circular DNA (cccDNA), which is arranged into a minichromosome and serves as a template for the production of viral mRNA, is formed during the viral life cycle (3). Chronic infection and a low rate of recovery are brought on by the extremely stable properties of cccDNA (4). Depending on the buildup of viral products, an infection might be directly cytopathic, non-cytopathic, or a combination of both in a particular patient (5).

HBV infections can range from asymptomatic infections or moderate illnesses to severe or infrequently fatal fulminant hepatitis. They can also be acute or chronic (6). Persistent HBV infection, which is defined as the presence of detectable hepatitis B surface antigen (HBsAg) in the blood or serum for more than six months, with or without concurrent active viral replication and signs of hepatocellular injury and inflammation, is a spectrum of diseases. One important determinant of the risk of persistent infection is age. Following an acute infection, chronicity is widespread (90%) in neonates and young children under five years old (20–60%), while it is rare (<5%) in adults (7).

One of the main causes of liver illness is still the hepatitis B virus (HBV), which also raises the risk of late-stage complications such as cirrhosis, hepatocellular carcinoma, and chronic hepatitis (8). Recently, significant progress has been achieved in the development of a limited HBV treatment that results in the permanent elimination of HBV DNA and HBsAg (a cure), which may offer the means of eradicating HBV as a threat to public health (9). Significant advancements in the efficacy of therapy have been made in the last few years due to a major improvement in our understanding of the epidemiology and natural history of HBV infection (10). In terms of reducing HCC incidence, decompensation, and mortality, as well as enhancing histology and inhibiting viral replication, the NA has demonstrated a strong safety profile and excellent efficacy (11).

The Global Health Sector Strategy on Viral Hepatitis was established by the World Health Assembly in 2016 to eradicate the disease by 2030 (12). A growing body of molecular, clinical, and financial evidence indicates that early antiviral treatment initiation could prevent HCC and save many lives at a very low cost (13). cccDNA is seldom eliminated by current antiviral treatments (4).

Establishing a functional cure, which is characterized by either the disappearance of hepatitis B surface antigen, undetectable serum HBV DNA levels, or seroconversion to hepatitis B surface antibody, has been the focus of recent antiviral treatment trials for chronic hepatitis B (14). By ensuring that everyone has equitable access to safe, cost-effective, and preventive diagnosis, treatment, and care of the highest caliber, FMOH hopes to eradicate viral hepatitis as a public health hazard in Ethiopia (15).

In close coordination with the Federal Ministry of Health (FMoH), the EthNoHep group initiated an HBV scale-up treatment program in Ethiopia. To begin treatment, it is based on four factors. Clinically confirmed cirrhosis, APRI >0·7, ALT >40 U/L, and an HBV viral load >2000 IU/mL are among these requirements. HCC in first-degree relatives is also required (16).

Globally, infections with the hepatitis B virus are a leading cause of infectious disease mortality and have become a significant public health concern(17). The burden of Hepatitis B virus (HBV) among chronic liver disease patients can vary significantly based on geographical location, prevalence of HBV in the general population, vaccination rates, and access to healthcare(1).

Globally, more than 2 billion individuals are currently infected with HBV, and 350 million of them are chronic carriers of the virus who have a high risk of dying from primary hepatocellular carcinoma, cirrhosis, and active hepatitis(18). The sub-Saharan region has one of the highest rates of hepatitis B surface antigen (HBsAg) carriers in the world, with a general population prevalence of 5–20%(19). In this region, the burden of HBV among chronic liver disease patients is likely to be substantial(20).

The prevalence of HBV among CLD patients significantly varies across regions in sub-Saharan Africa (21). The magnitude was 10.6% in Mbale Regional Referral Hospital, Uganda (22), 42.9 % in Ghana (23), and 71% in Nigeria (24). Ethiopia falls among the epidemiological category of regions where the prevalence of hepatitis B infection is classified as hyper endemic, with a prevalence ranging from 8 to 12% (25). Evidence in Ethiopia revealed that the magnitude of HBV among CLD patients ranges from 34.8 to 79.5% (26, 27).

Various sociodemographic, medical, surgical, obstetric, cultural, and behavioral factors were associated with an increased risk of HBV infection among chronic liver death patients. Some of the factors were middle age, male sex, marriage, living in a rural area, having fewer educational qualifications, smoking, having HBsAg-positive household contacts, having a family history of HBV, having undergone surgery, having received blood transfusions, dental operations in the past and traditional medical procedures (26-29).

The majority (95%) of HBV infections in immunocompetent adults are self-limited and resolve without treatment (6). However, the virus may cause cirrhosis, and liver cancer, which are the major causes of death, morbidity, and financial hardship (30). Evidence revealed that the pooled prevalence of cirrhosis was 4.1% in the primary care setting or general population and 12.7% in referral or tertiary care facilities in sub-Saharan Africa regions (31).

Worldwide, the most common cause of cirrhosis and liver cancer is chronic hepatitis B virus infection (32). In 2019, an estimated 331,000 people died from HBV-related cirrhosis, and around 192,000 people died from HBV-related liver cancer (32). In 2019, an estimated 331,000 people died from HBV-related cirrhosis, with another 192,000 dying from HBV-related liver cancer 32. Between 1990 and 2019, HBV-related mortality rose by 5.9%, and 2.9% between 2015 and 2019(33). According to a Global Burden of Disease study in 2010, cirrhosis mortality increased in sub-Saharan Africa between 1980 and 2010 and it was primarily due to hepatitis B (34%) (34).

The progression of patients with chronic hepatitis B to cirrhosis and HCC is influenced by various factors such as age over 40, male gender, presence of cirrhosis, family history of HCC, Asian and African racial backgrounds, genetic diversity, high levels of HBV replication during follow-up, HBV genotype, HDV, HCV, HIV concurrent infections, heavy alcohol consumption, aflatoxin, smoking, diabetes, and obesity (35).

Evidence in Ethiopia revealed that around 36.7% of chronic liver diseases were attributed to chronic hepatitis B infections(36). Other evidence showed that HBV is the most common cause of chronic liver disease, accounting for 40% of cases (37) Chronic liver disease is responsible for 12% of hospital admissions and 31% of fatalities in medical wards in Ethiopian hospitals(37).

With prioritized targets, interventions, and innovations, the World Health Organization intends to eradicate chronic viral hepatitis B as a hazard to public health by 2030 (38). Global plans to eliminate HBV infection include expanding population-level testing and treatment, stepping up HBV prevention interventions, and implementing universal HBV vaccination (39). The most effective method of eradicating illnesses linked to HBV is vaccination-based HBV infection prevention. A combination of passive immunization with hepatitis B immunoglobulin (HBIG) and active immunization with the HBV vaccine provides the best efficacy in preventing HBV infection(40).

Understanding the magnitude of the Hepatitis B virus and its associated factors among chronic liver disease patients is very important in assessing the burden of the disease and developing effective prevention and control strategies in Ethiopia. However, data on the magnitude of HBV and its associated factors among chronic liver disease patients is scarce in the study area as well as in Ethiopia at large. Therefore, this study was intended to assess the magnitude of HBV and its associated factors among chronic liver disease patients admitted to the medical ward at Sheikh Hassen Yabare Comprehensive Specialized Hospital, Jigjiga, Ethiopia.

# Materials and method

## Study Area

The study was conducted at the Sheikh Hassen Yabare Comprehensive Specialized Hospital, which is the first and largest specialized teaching hospital in the Somali Region State of Ethiopia It is at the center of Jigjiga city, which is located 610 km away from Addis Ababa in the eastern part of Ethiopia, and it lies at an altitude of 5361 feet (1634 meters) above sea level. It is expected to cover an estimated population of more than seven million people living in the region and neighboring districts of the Oromia region. The hospital also receives referrals from large parts of neighboring countries, like Somaliland and Puntland. The hospital provides health services at the inpatient and outpatient levels as a referral hospital for more than 7 million populations in the eastern part of the country. Under the Department of Internal Medicine, it has general medical wards with a total of 81 beds and chronic follow-up (41).

## Study design and period

## Hospital-based cross-sectional study design was conducted using a retrospective medical chart review. The study was conducted from 1/5/2024 –30/5/2024

## Source population

All chronic liver disease patients who had received medical care at Sheikh Hassen Yabare Comprehensive Specialized Hospital were the source population.

## Study population

## All selected clinically diagnosed chronic liver disease patients who had received medical care at Sheikh Hassen Yabare Comprehensive Specialized Hospital from 1/1/2020 to 1/12/ 2024 were the study population.

## Inclusion criteria

All chronic liver disease patients aged≥ 18 years and who had received medical care at Sheikh Hassen Yabare Comprehensive Specialized Hospital from 1/1/2020 to 1/12/ 2024.

**Exclusion criteria**

All patient charts with incomplete documentation of HBV status were excluded. In addition, medical charts having more than 20% of incomplete variables were excluded.

## Sample size Determination

The sample size was calculated using a single population proportion (p) among chronic liver disease patients.

**n=**$\frac{\left( \boldsymbol{Z}\frac{\boldsymbol{\alpha}}{\boldsymbol{2}} \right)\boldsymbol{2}\boldsymbol{\times}\boldsymbol{P}\left( \boldsymbol{1}\boldsymbol{-}\boldsymbol{P} \right)}{\boldsymbol{d}^{\boldsymbol{2}}}$

Where n= sample population for n>10,000

P = prevalence of HBV surface antigen on CLD(20.4%)(42)

d = marginal error (0.05)

Z (α/2) = the reliability coefficient of 95% i.e. 1.96

By using this formula the calculated sample size was 249

## Sampling technique and procedure

MRNs of CLD patients were collected from 1/1/2020 to 1/12/ 2024, from the Health Management Information System (HMIS) logbook of Sheikh Hassen Yabare Comprehensive Specialized Hospital. A total of 386 MRN were collected from 1/1/2020 to 1/12/ 2024. A representative sample was selected by a simple random sampling method using computer generated methods.

## Data collection method

The data collection tool was adapted from previous studies done in Ethiopia (26, 27). The tool contains sociodemographic characteristics, medical-related variables, surgical variables, obstetric and reproductive-related variables, and cultural and behavioral characteristics. First, the tool was entered into Kobo Toolbox software for data collection by the principal investigator.

Then, one-day training was provided for four medical interns (data collectors) by the principal investigator. MRNs of all chronic liver disease patients who had received medical care at Sheikh Hassen Yabare Comprehensive Specialized Hospital from 1/1/2020 to 1/12/ 2024, were retrieved from the Health Management Information System (HMIS) log Book of Sheikh Hassen Yabare Comprehensive Specialized Hospital. Then, medical charts were selected by the computer-generated random method and drawn from the medical chart office. The Sheik Hassen Yabare Comprehensive Specialized Hospital's main laboratory tested the HBsAg using a rapid test kit (Henso Medical (HANGZHOU) Co. LTD, China) in accordance with manufacturer protocol and standards. The safety and specimen handling precautions were properly followed during the tests.

Finally, the data collectors collect the data using the Kobo Toolbox software. The investigator managed the data collection procedure and evaluated the tool's completeness using Kobo Toolbox software.

## Study Variables

**Dependent variables**

- Hepatitis B virus sero-status (positive/negative)

**Independent variables**

- Sociodemographic variable; Age, sex, marital status, occupational status, residence
- Microsurgical variable; history of hospital admission, blood transfusion history, history of surgical procedure, dental extraction
- Cultural and behavioral variables; ear piercing, nose piercing, contact with jaundiced patients,
- sexual and obstetric variables; multiple sexual partners, abortion, sexually transmitted disease

## Operational definition

Hepatitis B virus serostatus can be seropositive or negative**.** Sero-positive HBV is defined as a positive HBsAg test, whereas Sero-negative HBV is defined as a negative HBsAg test (7).

**Multiple sexual partners**: individuals who have more than one sex partner (43)

## Contact history for a jaundiced person: a person who has a history of contact with a known HBsg-positive individual (44).

## Data Quality Control

To ensure the quality of data, one day of training was provided for four medical interns (data collectors) by the principal investigator on the objective of the study, the content of the questionnaire, and techniques of data collection. Before the actual data collection, a pre-test was conducted on 5% of the sample (12 cards) at Karamara Hospital. The collected data was checked continuously for completeness and consistency by the principal investigator daily.

## Data analysis and processing

The data were collected using Kobo tool box, for validation and cleaning and exported into SPSS version 29.0 for analysis, using custom syntax for data re-coding and diagnostics. Descriptive statistics were carried out, and the results were presented in tables and figures. Binary logistic regression was employed to identify statistically significant factors for HBV. In binary logistic regression, both bi-variable and multivariable analyses were done. Variables with a p-value of <0.25 in bi-variable logistic regression were selected as candidate variables for the final model (multivariable logistic regression model). Model fitness was checked by the Hosmer-Lemeshow goodness of test (P-value = 0.224) and multi-collinearity between the explanatory variables was checked using the variance inflation factor (VIF>10). In multivariable logistic regression, the backward stepwise method was used to select significant variables, and statistical significance was considered at P<0.05. An adjusted odd ratio (AOR) with a 95% confidence interval was used to measure the strength of the association.

## Ethical Considerations

Ethical approval for the research was obtained from the Jigjiga University Research and Ethics Review Board (JJURERB0056/2024). Permission was acquired from the hospitals to extract data from patient medical records, all the patients’ data were anonymized and the data from the medical records is held with strong Confidentiality.

# RESULTS

# Sociodemographic characteristics of study participants

This study included a total of 249 study participants. The age of study participants ranges from 18 to 80 years, with a median age and standard deviation (SD) of 38±15.85 years. Nearly one-third of the study participants (31.7%) were in the age group of 28–37 years. Around two-thirds, 176 (70.7%) of study participants were male. Nearly half (49.8%) of study participants were from urban areas (Table 1).

Table 1: Sociodemographic characteristics of chronic liver disease patients who had received medical care at Sheik Hassen Yabare Comprehensive Specialized Hospital from 1/1/2020 to 1/12/ 2024

| **Characteristics** | **Frequency (n)** | **Percent (%)** |
| --- | --- | --- |
| **Age (in years)** |  |  |
| 18-27 | 45 | 18.1 |
| 28-37 | 79 | 31.7 |
| 38-47 | 48 | 19.3 |
| 48-57 | 31 | 12.4 |
| ≥58 | 46 | 18.5 |
| **Sex** |  |  |
| Male | 176 | 70.7 |
| Female | 73 | 29.3 |
| **Residency** |  |  |
| Urban | 124 | 49.8 |
| Rural | 125 | 50.2 |

## Medical, surgical, and culturally related characteristics of study participants

Regarding medical-related characteristics, nearly one-third (32.9%) of study participants had a history of hospital admission. About one-fifth of the study participants (52, 20.9%) had a history of dental extraction. Around one-third (32.5%) of the study participants had a history of blood transfusion. Concerning culturally related characteristics, about 36 (14.5%) of study participants had a history of ear-piercing practices, while around 5 (2%) of study participants had a history of nose-piercing practices. Approximately 105 (42.2%) of study participants had contact history with jaundice patients (Table 2).

Table 2: Medical, surgical, and cultural-related characteristics of chronic liver disease patients who had received medical care at Sheik Hassen Yabare Comprehensive Specialized Hospital from 1/1/2020 to 1/12/ 2024.

| **Variables** | **Frequency (n)** | **Percent (%)** |
| --- | --- | --- |
| **Hospital admission history** |  |  |
| Yes | 82 | 32.9 |
| No | 167 | 67.1 |
| **History of blood transfusion** |  |  |
| Yes | 81 | 32.5 |
| No | 168 | 67.5 |
| **History of dental extraction** |  |  |
| Yes | 52 | 20.9 |
| No | 197 | 79.1 |
| **History of surgical procedure** |  |  |
| Yes | 45 | 18.1 |
| No | 204 | 81.9 |
| **History of ear piercing practice** |  |  |
| Yes | 36 | 14.5 |
| No | 213 | 85.5 |
| **History of nose piercing practice** |  |  |
| Yes | 5 | 2 |
| No | 244 | 98 |
| **Contact history to jaundice person** |  |  |
| Yes | 105 | 42.2 |
| No | 144 | 57.8 |

## Sexual and obstetric-related characteristics of study participants

Regarding obstetric characteristics, approximately one-fourth (19, or 25.67%) of women had a history of abortion. The majority (92%) of study participants had no history of sexually transmitted infections (STI) and about 36 (14.5%) of study participants had a history of multiple sexual partners (Table 3).

Table 3: Sexual and obstetric-related characteristics of chronic liver disease patients who had received medical care at Sheik Hassen Yabare Comprehensive Specialized Hospital from 1/1/2020 to 1/12/ 2024

| **Characteristics** | **Frequency(n)** | **Percent (%)** |
| --- | --- | --- |
| **History of abortion (n=73)** |  |  |
| Yes | 19 | 25.67 |
| No | 54 | 73.97 |
| **History of STI** |  |  |
| Yes | 20 | 8 |
| No | 229 | 92 |
| **Having multiple sexual partners** |  |  |
| Yes | 36 | 14.5 |
| No | 213 | 85.5 |

## Magnitude of HBV among clinically diagnosed chronic liver disease patients

The magnitude of HBV among clinically diagnosed chronic liver disease patients was 81/249 (32.53%) ( 95%CI = 26.9-38.6%) (Figure 1).

Figure 1: Magnitude of HBV among clinically diagnosed chronic liver disease patients who had received medical care at Sheik Hassen Yabare Comprehensive Specialized Hospital in 2024.

## Factors associated with HBV among clinically diagnosed chronic liver disease patients

Binary logistic regression was used to identify statistically significant factors associated with HBV. Bivariable logistic regression was done to identify candidate variables for multiple logistic regressions. Variables such as having a history of hospital admission, dental extraction, surgery, blood transfusion, contact history with jaundice patients, sex, and residency were significant at a p-value of <0.25 in bivariable logistic regression. In multivariable logistic regression, the backward stepwise method was used to select significant variables. Finally, four variables, namely male sex, having a history of hospital admission, blood transfusion, and contact history with jaundice patients, were significantly associated with HBV in clinically diagnosed chronic liver disease patients.

Chronic liver disease patients who had a history of hospital admission were 3.60 times more likely to have HBV infection as compared to their counterparts (AOR = 3.60, 95% CI = 1.84–7.04). In addition, chronic liver disease patients who had a history of blood transfusion were 2.22 times more likely to develop HBV as compared to their counterparts (AOR = 2.22, 95CI = 1.13–4.37). Furthermore, the likelihood of developing HBV was 2.65 times higher in male chronic liver disease patients as compared to female patients (AOR=2.65, 95CI=1.18, 5.96). Moreover, chronic liver disease patients who had a history of contact with patients with jaundice signs and symptoms were 4.52 times more likely to develop HBV as compared to their counterparts (AOR=4.52, 95CI=2.34, 8.72) (Table 4).

Table 4: shows the results of multivariable logistic regression for the association between HBV infection and independent variable among chronic liver disease patients who had received medical care at Sheik Hassen Yabare Comprehensive Specialized Hospital from 1/1/2020 to 1/12/ 2024.

| **Variable** | **HBV** | | **COR (95CI %)** | **AOR(95%CI)** |
| --- | --- | --- | --- | --- |
|  | **Positive** | **Negative** |  |  |
| **Sex** |  |  |  |  |
| Male | 70(86.4) | 106(63.1%) | 3.72(1.83,7.56) | 2.65(1.18, 5.96)* |
| Female | 11(13.6%) | 62(36.9%) | 1 | 1 |
| **History of dental extraction** |  |  |  |  |
| Yes | 27(33.3%) | 25(14.9%) | 2.86(1.52,5.35) | 1.89(0.87, 4.10) |
| No | 54(66.7%) | 143(85.1%) | 1 | 1 |
| **History of hospital admission** |  |  |  |  |
| Yes | 49(60.5%) | 33(19.6%) | 6.26(3.48,11.25) | 3.60(1.84, 7.04)* |
| No | 32(39.5%) | 135(80.4%) | 1 | 1 |
| **History of blood transfusion** |  |  |  |  |
| Yes | 43(53.1%) | 38(22.6%) | 3.87(2.19,6.82) | 2.22(1.13,4.37)* |
| No | 38(46.9%) | 130(77.4%) | 1 |  |
| **Contact history to Jaundice** |  |  |  |  |
| Yes | 59 (72.8%) | 46(27.4%) | 7.11(3.92,12.90) | 4.52(2.34, 8.72)* |
| No | 22 (27.2%) | 122(72.6%) | 1 | 1 |
| **Residency** |  |  |  |  |
| Urban | 36(44.4%) | 88(52.4%) | 1 | 1 |
| Rural | 45(55.6%) | 80(47.6%) | 1.37(0.80,2.34) | 1.91(0.99,3.70) |
| **History of surgery** |  |  |  |  |
| Yes | 19(23.5%) | 26(15.5%) | 1.67(0.86,3.24) | 1.62(0.70,3.72) |
| No | 62(76.5%) | 142(84.5%) | 1 | 1 |

* Indicates statically significant in multivariable logistic regression with p-value <0.05

# DISCUSSION

The magnitude of HBV among clinically diagnosed chronic liver disease patients was 81/249 (32.53%) ( 95%CI = 26.9-38.6%). This finding was in line with a study finding in Iran (38%) (45), the University of Gondar Comprehensive Specialized Hospital (34.8%) (26), and Public Hospitals in Addis Ababa (35.8%) (46).

The findings of this study were lower than those of study findings in South India (57%) (47), Albania (70%) (48), Ghana (42.9%) (23), and Nigeria (50%  and 71% of HBV in liver cirrhosis and hepatocellular carcinoma patients, respectively) (24). The variation might be because of differences in healthcare access and diagnostic capabilities between the two regions, which may lead to variations in the identification and reporting of HBV cases among chronic liver disease patients. In addition, there may be genetic variations in susceptibility to HBV infection or the progression of HBV-related liver disease between populations. Furthermore, the difference might be due to variations in study periods. Currently,  more attention is given to HBV in Ethiopia, and there is an increase in the HBV vaccination coverage. This condition might decrease the magnitude of HBV among CLD patients as compared to the previous studies in Albania, Nigeria, and India(49-51).

The finding of this study was also lower than a study finding in Hawassa University Comprehensive Specialized Hospital (79.5%) (27). The disparity across studies could be explained by differences in regional awareness, availability to preventative services, and health-seeking behaviors between Jigjiga and Hawassa, which can influence who is tested and when. Together, these characteristics indicate that the higher prevalence in Hawassa may be related to more risk-driven screening, whereas the lower rate in Jigjiga indicates a broader but less risk-concentrated sample.

On the other hand, this finding was higher than study findings in the Government Medical College & Rajindra Hospital, India (26%) (52), Yemen (24.1%) (53), and in Mbale Regional Referral Hospital, Uganda (10.6%) (22). This may be due to the smaller sample sizes in the previous studies, which were 100, 108, and 104 in India, Yemen, and Uganda, respectively, as compared to this study (n = 249).  In addition, disparities in healthcare infrastructure, including access to screening, diagnosis, and treatment for HBV, could contribute to variations in prevalence rates. Ethiopia may have limited resources and healthcare facilities compared to Uganda, leading to a higher burden of undiagnosed and untreated HBV cases. Furthermore, variations in cultural practices, beliefs, awareness of the routes of HBV transmission, efforts made by health professionals to implement universal precautions, and preliminary benefits due to the initiation of national immunization programs in other countries could contribute to differences in prevalence rates between Ethiopia and previous studies in Yemen and Uganda.

In this study, chronic liver disease patients with a history of blood transfusion were more likely to develop HBV infection as compared to their counterparts (AOR = 2.22, 95CI = 1.13–4.37). This finding was supported by a study in Accra, Ghana (52) ,and Hawassa University Comprehensive Specialized Hospital (27). The association could be explained by the fact that patients who received blood transfusions many years ago may have been exposed to HBV at a time when screening and testing were not as rigorous as they are today. As a result, they may have developed chronic liver disease over time due to the long-term effects of HBV infection acquired through blood transfusion

This study revealed that having a history of hospital admission was significantly associated with HBV infection among chronic liver disease patients (AOR = 3.60, 95% CI = 1.84–7.04). This might be due to nosocomial transmission. Hospital settings can be a source of HBV transmission due to the potential for exposure to contaminated blood or bodily fluids. Patients with chronic liver disease who have been hospitalized multiple times may have had increased opportunities for exposure to HBV within healthcare facilities. In addition, patients with chronic liver disease often require frequent medical interventions, such as surgeries, endoscopies, or other invasive procedures. These procedures can pose a risk of HBV transmission if proper infection control measures are not followed, leading to an increased likelihood of acquiring the virus during hospital admissions. Furthermore, in some cases, medical equipment may not be adequately sterilized between uses, increasing the risk of cross-contamination and HBV transmission among patients.

This study also found that having a contact history with jaundiced patients was significantly associated with HBV infection (AOR=4.52, 95CI=2.34, 8.72). This finding was in agreement with a study finding in the University of Gondar Comprehensive Specialized Hospital (26). This could be justified by the direct transmission of the virus from patients with jaundice signs and symptoms. Jaundice is a common symptom of acute HBV infection, and individuals with jaundice are likely to have high levels of the virus in their blood, making them more infectious.

Finally, this study demonstrated that male sex was significantly associated with HBV infection (AOR=2.65, 95%CI=1.18, 5.96). This finding was supported by studies conducted in the Taiwanese population (54), Turkey (55), and the University of Gondar Comprehensive Specialized Hospital (26). This could be explained by high-risk behaviors in males as compared to females. Men are more likely to engage in high-risk behaviors that increase their risk of HBV transmission, such as unprotected sex and intravenous drug use. These behaviors can lead to a higher rate of HBV infection among male individuals. In addition, men may be more likely to work in occupations with a higher risk of exposure to HBV, which increases their risk of infection.

# Limitations of the study

# The nature of the retrospective data is prone to missing data, and this could be the limitation of the study. In addition to that, this study did not assess some important behavioral and cultural factors such as the history of uvuloctomy, parenteral drug abuse, history of tattooing, delivery history by traditional birth attendants, traditional body piercing, and sharing sharp instruments. In addition, this study was conducted in a single center and included a small number of study subjects, which may affect its generalizability. Furthermore, chronic liver disease patients with occult HBV infection were not identified due to its retrospective design.

# Conclusion

The magnitude of HBV among chronic liver disease patients was high in Sheik Hassen Yabare Comprehensive Specialized Hospital. Being male, having a history of hospital admission, blood transfusion, and having a contact history with jaundice patients were significantly associated with HBV in patients with chronic liver disease. Hospitals should follow strict blood transfusion safety protocols and implement infection control measures to reduce HBV transmission. Screening patients with a history of hospital admission, blood transfusion, or jaundice contact can facilitate early diagnosis and treatment. Increase public awareness about HBV transmission modes through mass media and campaigns. Future studies should evaluate unaddressed behavioral and cultural factors using a multi-center site, a large number of participants, and a prospective design.

**Acknowledgment**

The authors express their gratitude to everybody who contributed to this original article at any step.

**Conflict of interest**

The authors sustain no conflict of interest.

**Funding statement**

The authors received no financial support for this research

**Data availability statement**

The data that support the findings of this study are available from the corresponding author upon reasonable request.

**ORCID**

Ramadan Budul Yusuf: https://orcid.org/0009-0001-0379-5545

Ahmed Mohammed Ibrahim <https://orcid.org/0000-0002-3493-8473>

Supporting information

S1 File. Data used in the analysis for this study.(CSV format)

# REFERENCE

1.	Zhao R-H, Shi Y, Zhao H, Wu W, Sheng J-F. Acute-on-chronic liver failure in chronic hepatitis B: an update. Expert review of gastroenterology & hepatology. 2018;12(4):341-50.

2.	Yuen MF, Chen DS, Dusheiko GM, Janssen HLA, Lau DTY, Locarnini SA, et al. Hepatitis B virus infection. Nat Rev Dis Primers. 2018;4:18035.

3.	Valaydon ZS, Locarnini SA. The virological aspects of hepatitis B. Best Pract Res Clin Gastroenterol. 2017;31(3):257-64.

4.	Tsukuda S, Watashi K. Hepatitis B virus biology and life cycle. Antiviral Res. 2020;182:104925.

5.	Zhang YY, Hu KQ. Rethinking the pathogenesis of hepatitis B virus (HBV) infection. J Med Virol. 2015;87(12):1989-99.

6.	who. GUIDELINES FOR THE PREVENTION, CARE AND TREATMENT OF PERSONS WITH CHRONIC HEPATITIS B INFECTION MARCH 2015. 2015:43;166.

7.	Liang TJ. Hepatitis B: the virus and disease. Hepatology. 2009;49(5 Suppl):S13-21.

8.	İnan N, Tabak F. Hepatitis B Virus: Biology and Life Cycle. Viral Hepatit Dergisi. 2015;21(1):1-7.

9.	Howell J, Seaman C, Wallace J, Xiao Y, Scott N, Davies J, et al. Pathway to global elimination of hepatitis B: HBV cure is just the first step. Hepatology. 2023;78(3):976-90.

10.	Marcellin P. Hepatitis B and hepatitis C in 2009. Liver Int. 2009;29 Suppl 1:1-8.

11.	Broquetas T, Carrión JA. Past, present, and future of long-term treatment for hepatitis B virus. World J Gastroenterol. 2023;29(25):3964-83.

12.	Waheed Y, Siddiq M, Jamil Z, Najmi MH. Hepatitis elimination by 2030: Progress and challenges. World J Gastroenterol. 2018;24(44):4959-61.

13.	Lim YS, Kim WR, Dieterich D, Kao JH, Flaherty JF, Yee LJ, et al. Evidence for Benefits of Early Treatment Initiation for Chronic Hepatitis B. Viruses. 2023;15(4).

14.	Kim SW, Yoon JS, Lee M, Cho Y. Toward a complete cure for chronic hepatitis B: Novel therapeutic targets for hepatitis B virus. Clin Mol Hepatol. 2022;28(1):17-30.

15.	FMOH. NATIONAL STRATEGIC PLAN FOR PREVENTION AND CONTROL

OF VIRAL HEPATITIS IN ETHIOPIA, 2021 – 2025. 2021:14;49.

16.	Johannessen A, Gebremedhin LT, Desalegn H. New WHO hepatitis B treatment guidelines: look to Ethiopia. Lancet Glob Health. 2022;10(12):e1711-e2.

17.	Alter MJ. Epidemiology of hepatitis B in Europe and worldwide. Journal of Hepatology. 2003;39:64-9.

18.	Kane M. Global programme for control of hepatitis B infection. Vaccine. 1995;13 Suppl 1:S47-9.

19.	Kiire CF. Hepatitis B infection in sub-Saharan Africa. The African Regional Study Group. Vaccine. 1990;8 Suppl:S107-12; discussion S34-8.

20.	Surial B, Wyser D, Béguelin C, Ramírez‐Mena A, Rauch A, Wandeler G. Prevalence of liver cirrhosis in individuals with hepatitis B virus infection in sub‐Saharan Africa: Systematic review and meta‐analysis. Liver international. 2021;41(4):710-9.

21.	Kafeero HM, Ndagire D, Ocama P, Kudamba A, Walusansa A, Sendagire H. Prevalence and predictors of hepatitis B virus (HBV) infection in east Africa: evidence from a systematic review and meta-analysis of epidemiological studies published from 2005 to 2020. Arch Public Health. 2021;79(1):167.

22.	Bewala F. Prevalence of hepatitis B and C viral infections in patient's tentatively diagnosed with liver diseases at Mbale Regional Referral Hospital: Makerere University; 2022.

23.	Blankson A, Wiredu E, Gyasi R, Adjei A, Tettey Y. Sero-prevalence of hepatitis B and C viruses in cirrhosis of the liver in Accra, Ghana. Ghana Medical Journal. 2005;39(4):132.

24.	Ola S, Odaibo G, Olaleye O. HCV and HBV infection in Nigerian patients with liver cirrhosis and hepatocellular carinoma. 2004.

25.	FMOH. ethiopian National strategy for prevention and control of viral hepatitis. 2016:11;59.

26.	Assefa D, Abay Z, Tsegaye T, Ahmed E, Diress A. Seroprevalence of Hepatitis B virus and Associated factors among adult Chronic liver disease patients at University of Gondar Comprehensive Specialized Hospital, Northwest Ethiopia. International Internal Medicine Journal. 2023;1(6):272-80.

27.	Deregasso BL, Ababiya RH, Rediet Ajebu Nurfeta3, Ababiya TH, Tema LA, Teka BG, et al. Hepatitis B Prevalence and Risk Factors in Chronic Liver Disease Patients at Hawassa University Comprehensive Specialized Hospital, Sidama Region, Ethiopia. Science Journal of Clinical Medicine. 2024;13(1):1-8.

28.	Hamilton EM, Rassam W, Yan Y, Singh A, Ng SYA, Zhang J, et al. Correlates of chronic hepatitis B virus infection in the general adult population of China: Systematic review and meta-analysis. J Viral Hepat. 2023;30(6):470-88.

29.	Weldebrhan D, Berhe H, Tesfay Y. Risk Factors for Hepatitis B Virus Infection in North Ethiopia: A Case-Control Study. Hepat Med. 2023;15:79-91.

30.	Younossi ZM, Wong G, Anstee QM, Henry L. The Global Burden of Liver Disease. Clin Gastroenterol Hepatol. 2023;21(8):1978-91.

31.	Surial B, Wyser D, Béguelin C, Ramírez-Mena A, Rauch A, Wandeler G. Prevalence of liver cirrhosis in individuals with hepatitis B virus infection in sub-Saharan Africa: Systematic review and meta-analysis. Liver Int. 2021;41(4):710-9.

32.	Hsu YC, Huang DQ, Nguyen MH. Global burden of hepatitis B virus: current status, missed opportunities and a call for action. Nat Rev Gastroenterol Hepatol. 2023;20(8):524-37.

33.	GBD. Global, regional, and national burden of hepatitis B, 1990-2019: a systematic analysis for the Global Burden of Disease Study 2019. Lancet Gastroenterol Hepatol. 2022;7(9):796-829.

34.	Spearman CW, Sonderup MW. Health disparities in liver disease in sub‐Saharan Africa. Liver International. 2015;35(9):2063-71.

35.	Fattovich G, Bortolotti F, Donato F. Natural history of chronic hepatitis B: special emphasis on disease progression and prognostic factors. J Hepatol. 2008;48(2):335-52.

36.	Orlien SMS, Ismael NY, Ahmed TA, Berhe N, Lauritzen T, Roald B, et al. Unexplained chronic liver disease in Ethiopia: a cross-sectional study. BMC gastroenterology. 2018;18:1-12.

37.	Tesfaye BT, Feyissa TM, Workneh AB, Gudina EK, Yizengaw MA. Chronic Liver Disease in Ethiopia with a Particular Focus on the Etiological Spectrums: A Systematic Review and Meta-Analysis of Observational Studies. Can J Gastroenterol Hepatol. 2021;2021:8740157.

38.	who. Global health sector strategies on, respectively, HIV, viral hepatitis and sexually transmitted infections for the period 2022-2030. 2022:72;134.

39.	Nayagam S, Thursz M. Strategies for Global Elimination of Chronic HBV Infection: 2019 Update. Current Hepatology Reports. 2019;18(3):300-9.

40.	Chang MH, Chen DS. Prevention of hepatitis B. Cold Spring Harb Perspect Med. 2015;5(3):a021493.

41.	hospital S. <JJU, SHY CS Hospital Profile, Jan.2023.pdf>2023.

42.	Mengiste DA, Dirbsa AT, Ayele BH, Hailegiyorgis TT. Hepatitis B virus infection and its associated factors among medical waste collectors at public health facilities in eastern Ethiopia: a facility-based cross-sectional study. BMC Infectious Diseases. 2021;21(1):233.

43.	Umare A, Seyoum B, Gobena T, Haile Mariyam T. Hepatitis B Virus Infections and Associated Factors among Pregnant Women Attending Antenatal Care Clinic at Deder Hospital, Eastern Ethiopia. PLoS One. 2016;11(11):e0166936.

44.	Mohammed H, Eshetie A, Melese D. Prevalence of hepatitis B virus and associated risk factors among adults patients at Dessie referral and Kemise general hospitals in northeastern Ethiopia. Health Sci Rep. 2022;5(3):e659.

45.	Anvari FA, Alavian SM, Norouzi M, Mahabadi M, Jazayeri SM. Prevalence and molecular analysis of occult hepatitis B virus infection isolated in a sample of cryptogenic cirrhosis patients in iran. Oman Medical Journal. 2014;29(2):92.

46.	Ayele AG, Gebre-Selassie S. Prevalence and risk factors of hepatitis B and hepatitis C virus infections among patients with chronic liver diseases in public hospitals in Addis Ababa, Ethiopia. ISRN Tropical Medicine. 2013;2013:1-7.

47.	Saravanan S, Velu V, Kumarasamy N, Shankar EM, Nandakumar S, Murugavel KG, et al. The prevalence of hepatitis B virus and hepatitis C virus infection among patients with chronic liver disease in South India. International journal of infectious diseases. 2008;12(5):513-8.

48.	Kondili LA, Çuko L, Chionne P, Candido A, Madonna E, Dentico P, et al. Hepatitis B, C and Delta virus infections in Albanian patients with chronic liver disease: evaluation of possible changes during the last 10 years. European journal of gastroenterology & hepatology. 2010;22(2):167-71.

49.	Jaho J, Kamberi F, Mechili EA, Bicaj A, Carnì P, Baiocchi L. Review of Albanian studies suggests the need for further efforts to counteract significant hepatitis B virus prevalence. World journal of virology. 2024;13(4):93721.

50.	Ajuwon BI, Yujuico I, Roper K, Richardson A, Sheel M, Lidbury BA. Hepatitis B virus infection in Nigeria: a systematic review and meta-analysis of data published between 2010 and 2019. BMC Infect Dis. 2021;21(1):1120.

51.	Ray G. Current Scenario of Hepatitis B and Its Treatment in India. Journal of clinical and translational hepatology. 2017;5(3):277-96.

52.	Grewal US, Walia G, Bakshi R, Chopra S. Hepatitis B and C viruses, their coinfection and correlations in chronic liver disease patients: a tertiary care hospital study. International Journal of Applied and Basic Medical Research. 2018;8(4):204-9.

53.	Guneid AME, Gunaid AA, O'Neill AM, Zureikat NI, Coleman JC, Murray‐Lyon IM. Prevalence of hepatitis B, C, and D virus markers in Yemeni patients with chronic liver disease. Journal of medical virology. 1993;40(4):330-3.

54.	Wang AC, Geng J-H, Wang C-W, Wu D-W, Chen S-C. Sex difference in the associations among risk factors with hepatitis B and C infections in a large Taiwanese population study. Frontiers in Public Health. 2022;10:1068078.

55.	Ozer A, Yakupogullari Y, Beytur A, Beytur L, Koroglu M, Aydogan F. Risk factors of hepatitis B virus infection in Turkey: A population-based, case-control study: Risk Factors for HBV Infection. Hepatitis monthly. 2011;11(4):263.
