# Supplementary material for: Magnitude of Hepatitis B virus and associated factors among chronic liver disease patients admitted to the medical ward at Sheikh Hassen Yabare Comprehensive Specialized Hospital, Jigjiga, Ethiopia: a retrospective study
Source: PLoS One. 2025 Dec 30;20(12):e0338702. doi: 10.1371/journal.pone.0338702 (PMC12752937; doi:10.1371/journal.pone.0338702)
Supplement: Attachment — Submitted filename: PLOS_ONE_Review.docx [file pone.0338702.s006.docx]

# Review of Manuscript Against PLOS ONE Publication Criteria

This document reviews the provided manuscript based on the official PLOS ONE publication criteria. The assessment highlights strengths, areas requiring improvement, and overall eligibility for publication in PLOS ONE.

## ✅ Strengths (Meets Criteria)

- Original research: The manuscript presents primary data collected from chronic liver disease patients in Ethiopia.
- Clear methods and results: The study applies a retrospective design and includes regression analysis with confidence intervals.
- Relevance to health sciences: The work addresses the prevalence of HBV in chronic liver disease patients, which is of clinical and public health significance.
- Ethical scope: Patient details are anonymized, and the subject matter is consistent with ethical publication standards.

## ⚠️ Areas Needing Improvement (Potential Barriers)

- Scientific rigor: The methods section should describe study design, inclusion/exclusion criteria, diagnostic procedures, and statistical methods in detail.
- Novelty: PLOS ONE does not require high impact, but the manuscript should emphasize what is new compared to previous Ethiopian HBV studies.
- Data availability: Data must be deposited in a public repository (e.g., Dryad, Zenodo, Figshare). 'Available upon request' is not sufficient for PLOS ONE.
- Formatting: Tables and figures must follow PLOS ONE style. References should include DOIs and use correct formatting.
- Ethics statement: A detailed ethics approval statement (reviewing body, approval number, consent process) must be included.
- Language: The text requires editing for clarity, conciseness, and grammar to improve readability.

## 📋 Overall Assessment

The manuscript qualifies as original research and is therefore eligible for consideration by PLOS ONE. However, revisions are needed before submission:
1. Expand the Methods section and include Ethics approval details.
2. Deposit the dataset in a public repository with a DOI.
3. Strengthen the justification of novelty.
4. Revise references, tables, and figures to match journal style.
5. Proofread the text for clarity and grammar.
