# Supplementary material for: Magnitude of Hepatitis B virus and associated factors among chronic liver disease patients admitted to the medical ward at Sheikh Hassen Yabare Comprehensive Specialized Hospital, Jigjiga, Ethiopia: a retrospective study
Source: PLoS One. 2025 Dec 30;20(12):e0338702. doi: 10.1371/journal.pone.0338702 (PMC12752937; doi:10.1371/journal.pone.0338702)
Supplement: Attachment — Submitted filename: Response to reviewers.docx [file pone.0338702.s008.docx]

**Reviewers' comments:**

**Comments to the Author**

**Reviewer #1:** The methods require greater detail on sample selection, diagnostic procedures, and handling of missing data to ensure reproducibility. The manuscript also lacks a complete ethics approval statement and does not comply with PLOS ONE’s strict data availability policy; the dataset must be deposited in a public repository. In addition, the contribution of this study compared to previous Ethiopian work should be more clearly articulated, tables and references should be reformatted to meet journal style, and the language should be edited for clarity. Overall, the article has merit but requires minor revision to meet the journal’s standards.

**Response to Reviewer**

Dear reviewer, we acknowledge your insight,

- We have put the details of the diagnostic procedure
- We have mentioned in the exclusion criteria that incomplete records were excluded, and the issue of missing data was handled in that manner.
- We have also deposited the data into a public repository (Zenodo), and it can be accessed at (<https://doi.org/10.5281/zenodo.17264843>).
- The ethical approval statement was provided, and the detail was mentioned under the ethical consideration section.
- The tables and the figures were formatted to meet the journal’s style
- All the formatting and grammatical issues were addressed.

Reviewer #2:

1. The study relies on a rapid HBsAg test kit for HBV diagnosis. The potential for lower sensitivity and specificity of such kits compared to laboratory gold standards could lead to an underestimation of the true disease prevalence. This question should be acknowledged and discussed in the 'Limitations' section.

**Response to Reviewer**

Dear reviewer, we highly appreciate your insight,

We have addressed and included the potential for lower sensitivity and specificity compared to the gold standard in the limitations section.

2. The manuscript concludes that hospitalization is a risk factor for HBV infection. However, this interpretation overlooks the potential for reverse causality. It is plausible that severe HBV-related liver disease itself necessitates frequent hospital admissions. This explanation should be included in the Discussion.

**Response to Reviewer**

Dear reviewer, we highly appreciate your constructive insight. Since there were no HBV routine screenings on patients at admission, the issue of reverse causality could not be ruled out, and we have addressed it under the limitation section, with a recommendation of a prospective study in the future.

3. The manuscript lacks consistency in the presentation of statistical notations. For instance, confidence intervals are variously formatted as "95%CI" and "95CI" across the text. Similarly, the notation for p-values is not uniform. These terms must be standardized throughout the manuscript for clarity and professionalism.

**Response to Reviewer**

Dear reviewer, we highly appreciate your constructive insight. We have adjusted all the statistical notations per the comment, and all the terms were consistently written with clarity.

**Editor’s comments**

1. Expand the Methods section and include Ethics approval details.

- Dear editor, we have expanded the method section, including the diagnostic procedure and ethical approval details.

2. Deposit the dataset in a public repository with a DOI.

- We have also deposited the data into a public repository (Zenodo), and it can be accessed at (<https://doi.org/10.5281/zenodo.17264843>).

3. Strengthen the justification of novelty.

- This study was conducted in a population with distinct so**cio-cultural practices, healthcare access challenges, and epidemiological patterns.**

4. Revise references, tables, and figures to match journal style.

- All are revised to match the journal style.

5. Proofread the text for clarity and grammar.

- Thanks, we addressed all the formatting and grammatical issues.
