# Supplementary material for: Magnitude of Hepatitis B virus and associated factors among chronic liver disease patients admitted to the medical ward at Sheikh Hassen Yabare Comprehensive Specialized Hospital, Jigjiga, Ethiopia: a retrospective study
Source: PLoS One. 2025 Dec 30;20(12):e0338702. doi: 10.1371/journal.pone.0338702 (PMC12752937; doi:10.1371/journal.pone.0338702)
Supplement: Attachment — Submitted filename: Response to editor.docx [file pone.0338702.s009.docx]

**Editor’s comments**

Editor Comments:

1. Line 35: (HANGZHOU, Co. Ltd.); similarly, do it in the methods part

**Author’s response**: Thanks, dear editor, we have done so based on your suggestion on line 213.

2. Insert the assumptions and prevalence (P) into the sample size determination formula and show it.

**Author’s response**: We really appreciate your insightful suggestion, and we inserted the assumptions into the formula, line 193.

3. Line 170: The study was conducted from May 01, 2024, to May 30, 2024. Write lines 176, 181, 195,197, and 206 similar to line 170 (the date).

**Author’s response**: Dear editor, we have changed the date to the format you suggested for lines 170, 176,181, 196, 199, 209, 278, 291, 170,176,181,196,199,209,278,291,300, and 328, including the tables.

4. Line 205: Please write out the full form of MRNs. A sentence cannot start with an abbreviation.

**Author’s response**: Dear editor, we acknowledge your suggestions, and have written the full form in all lines starting with MRNs (Medical record numbers) for lines: 196,198, and 207.

5. Please ensure that all dates in each table are aligned with the above comment.

**Author’s response**: We really appreciate your insight and have fully aligned the dates per the comment you have given us.
